# Establishment and Characterization of an Empirical Biomarker SS/PV-ROC Plot Using Results of the UBC^®^ *Rapid* Test in Bladder Cancer

**DOI:** 10.3390/e22070729

**Published:** 2020-06-30

**Authors:** Peter Oehr, Thorsten Ecke

**Affiliations:** 1 Faculty of Medicine, Rheinische Friedrich-Wilhelms-Universität Bonn, 53113 Bonn, Germany; 2 Department of Urology, HELIOS Hospital, 15526 Bad Saarow, Germany

**Keywords:** predictive ROC curve, ROC curve, PV-ROC curve, SS-ROC curve, SS/PV-ROC plot, empirical, urinary bladder cancer

## Abstract

Background: This investigation included both a study of potential non-invasive diagnostic approaches for the bladder cancer biomarker UBC^®^ *Rapid* Test and a study including comparative methods about sensitivity–specificity characteristic (SS-ROC) and predictive receiver operating characteristic (PV-ROC) curves that used bladder cancer as a useful example. Methods: The study included 289 urine samples from patients with tumors of the urinary bladder, patients with non-evidence of disease (NED) and healthy controls. The UBC^®^ *Rapid* Test is a qualitative point of care assay. Using a photometric reader, quantitative data can also be obtained. Data for pairs of sensitivity/specificity as well as positive/negative predictive values were created by variation of threshold values for the whole patient cohort, as well as for the tumor-free control group. Based on these data, sensitivity–specificity and predictive value threshold distribution curves were constructed and transformed into SS-ROC and PV-ROC curves, which were included in a single SS/PV-ROC plot. Results: The curves revealed TPP-asymmetric improper curves which cross the diagonal from above. Evaluation of the PV-ROC curve showed that two or more distinct positive predictive values (PPV) can correspond to the same value of a negative predictive value (NPV) and vice versa, indicating a complexity in PV-ROC curves which did not exist in SS-ROC curves. In contrast to the SS-ROC curve, the PV-ROC curve had neither an area under the curve (AUC) nor a range from 0% to 100%. Sensitivity of the qualitative assay was 58.5% and specificity 88.2%, PPV was 75.6% and NPV 77.3%, at a threshold value of approximately 12.5 µg/L. Conclusions: The SS/PV-ROC plot is a new diagnostic approach which can be used for direct judgement of gain and loss of predictive values, sensitivity and specificity according to varied threshold value changes, enabling characterization, comparison and evaluation of qualitative and quantitative bioassays.

## 1. Introduction

In clinical practice for the detection of urinary bladder cancer, the confirmatory gold-standard procedure, cystoscopy, is invasive, costly and time consuming. Thus, there is interest in easy to perform rapid noninvasive bioassays at lower cost for detecting cancer disease in urinary samples from patients with suspected bladder cancer or for follow-up of the disease in bladder cancer patients.

To date, antigens determined by bioassays for urinary bladder cancer are not tumor specific. However, since bladder cancer tissue, compared to normal tissue, often expresses higher concentrations for those antigens, elevated levels can also be found in urine of bladder cancer patients, when compared to the levels of individuals without cancer. This enables the use of such assays for antigen determination in the diagnostics of urinary bladder cancer to a certain extent.

A new type of noninvasive urine-based tumor marker test for detection of urinary bladder cancer is the UBC^®^ *Rapid* Test (Urinary Bladder Cancer Antigen *Rapid* Test). This biomarker test is a visual point of care (POC) test, detecting antigen fragments of cytokeratin 8 and 18 from urine samples only qualitatively. These antigens can also be determined quantitatively using the qualitative POC UBC^®^ *Rapid* Test, combined with a photometric POC reader. With respect to quantitative assay determinations, cytokeratin levels are lower in low-grade tumors and benign urological diseases, compared to high-grade tumors [1,2]. Recent investigations including sensitivity–specificity characteristic (SS-ROC) curves gave evidence of utility for the UBC^®^ *Rapid* Test in detecting CIS (carcinoma in situ) and non-invasive high-risk tumors [3,4].

The aim of this investigation was to evaluate the quality of the non-invasive diagnostic approaches for the qualitative and quantitative bladder cancer biomarker UBC^®^ *Rapid* on the basis of tables and distribution curves for sensitivity, specificity and predictive values. Using the underlying study of bladder cancer as a useful example, the study was furthermore intended to access preliminary information about the utility of a newly developed graph consisting of both a sensitivity–specificity ROC SS-ROC and a predictive value ROC curve (PV-ROC), called a SS/PV-ROC plot. According to the present literature, there seems to be no publications on empirical PV-ROC-curves, and no SS/PV-ROC plot has been published to date.

## 2. Methods

The study was approved by the local Institutional Review Board of Medical Association Brandenburg (AS 147(bB)/2013).

In total, 289 urine samples were included in this prospective study. Clinical urine samples from 111 patients with tumors of the urinary bladder, 32 patients with non-evidence of disease (NED) and 146 healthy controls were used. Midstream urine was collected in a sterile plastic container and processed subsequently. Urine samples were analyzed by the UBC^®^ *Rapid* Test (concile GmbH, Freiburg/Breisgau, Germany).

All patients with confirmed bladder cancer underwent cystoscopy, bladder ultrasound and transurethral resection of bladder tumor in the case of abnormal findings. Exclusion criteria were any kind of mechanical manipulation (cystoscopy, transrectal ultrasound and catheterization) within 10 days prior to urine sampling. Other exclusion criteria were benign prostate enlargement, urolithiasis other tumor diseases; severe infections; and pregnancy. All these criteria could influence the test to yield false positive results. Table 1 illustrates the characteristics of all bladder cancer patients.

The UBC^®^ *Rapid* Test was performed by qualitative visual estimation of positive/negative results. Presently used POC-assays use qualitative immuno-chromatographic lateral flow assays which develop a concentration-dependent color reaction used as a threshold on a test line. A positive reaction is determined by subjective decision of human operators. The UBC^®^ *Rapid* Test in combination with a POC-reader system enables quantitative determination of a tumor-marker under POC-assay conditions. Photometric readers can transform the concentration-dependent color reaction into quantitative values and represent a new development including objective, quantitative evaluation of POC-assays.

Data for pairs of sensitivity/specificity as well as positive/negative predictive values were created by variation of test threshold values for the whole patient cohort as well as for the tumor-free control group. The cut-offs were only selected to cover the range of the biomarker test used in the study, in order to have several values for plotting the SS-ROC and the PV-ROC curves. There were no clinical selection criteria, or criteria concerning an optimal cut-off. Based on these data, sensitivity–specificity and predictive value distribution curves were constructed and transformed into SS-ROC and PV-ROC curves, and then drawn together on a single SS/PV-ROC plot. In addition, the values for sensitivity, specificity and predictive values of the qualitative POC assay were plotted, each as a single point, for direct assignment to both ROC curves, as well as for estimation of the qualitative test’s threshold values.

### Statistical Analysis

According to the tables, all statistical analyses were performed using R version 3.2.3 (R Core Team (2015) [5]. Data are presented descriptively using means and standard deviations for numerical variables and absolute and relative frequencies for categorical variables.

Data evaluation for the curves was conducted using Excel. True and false positive and true and false negative results of the qualitative and quantitative assays were calculated and applied for plotting distribution curves for sensitivity, specificity and positive and negative predictive values, as well as for SS-ROC and PV-ROC curves with respect to their corresponding set threshold values.

## 3. Results

Table 1 illustrates the data of the patients. Tumor stages pTa and pT1 include non-muscle-invasive (NMI) bladder cancer, while tumor stages pT2 and pT3 include muscle invasive (MI) bladder cancer; only CIS (carcinoma in situ) is a tumor type with a high risk of recurrence and progress. Grading of bladder cancer is stratified from high (G1) to low (G3) differentiation. European Organisation for Research and Treatment of Cancer (EORTC) risk is defined after the definition of the European Association of Urology [6,7]. Table 2 contains the description of study data. Clinical data were evaluated with respect to the UBC^®^ *Rapid* Test. Table 2 shows that pathological concentrations of the UBC^®^ *Rapid* Test are detectable in urine of bladder cancer patients. Pathological levels of the UBC^®^ *Rapid* Test in urine are higher in patients with bladder cancer in comparison to the control group.

**Figure 1:**
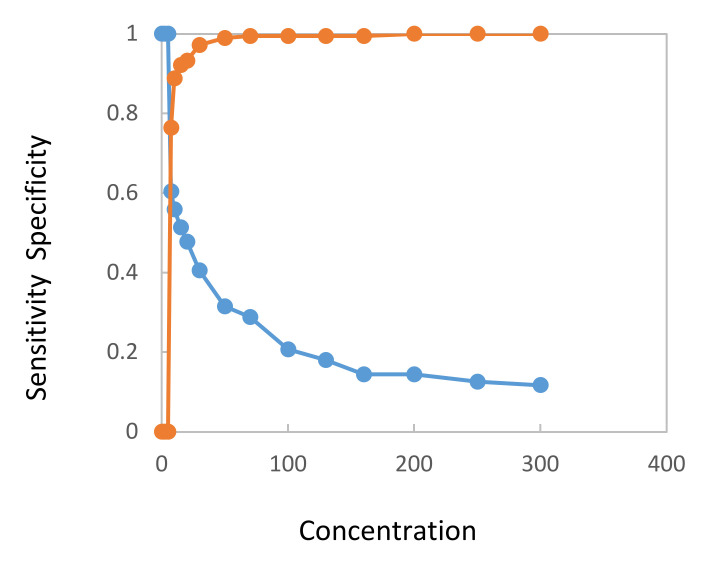
Distribution curves for sensitivity (blue line) and specificity (brown line) from setting various threshold values over the whole range of the UBC^®^ *Rapid* Test concentrations (µg/L).

**Figure 2:**
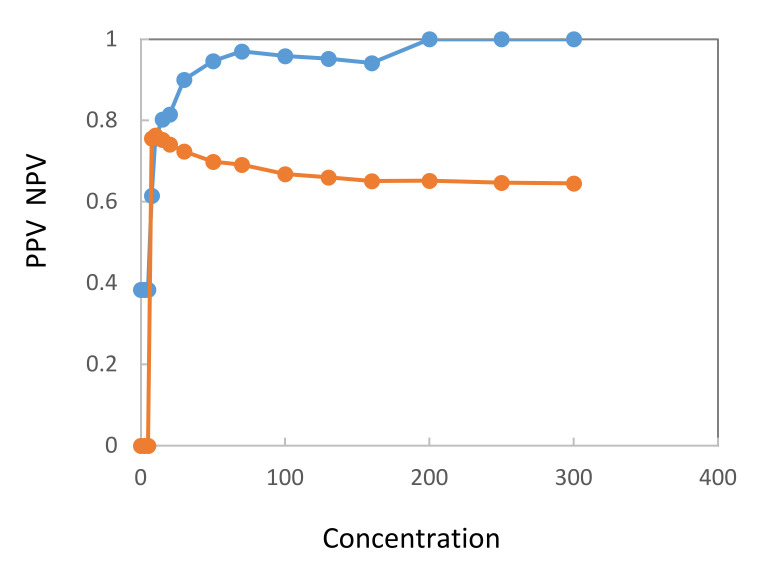
Distribution curves for positive (blue line) and negative (brown line) predictive values from setting various threshold values over the whole range of the UBC^®^ *Rapid* Test concentrations (µg/L).

Figure 1 and Figure 2 show concentration distribution curves calculated from values determined from samples of bladder cancer patients and cancer-free controls. Figure 1 refers to sensitivity and specificity, Figure 2 to PPV and NPV. Both figures confirm that the quantitative UBC^®^ *Rapid* Test can discriminate between bladder cancer patients and cancer-free controls.

**Figure 3:**
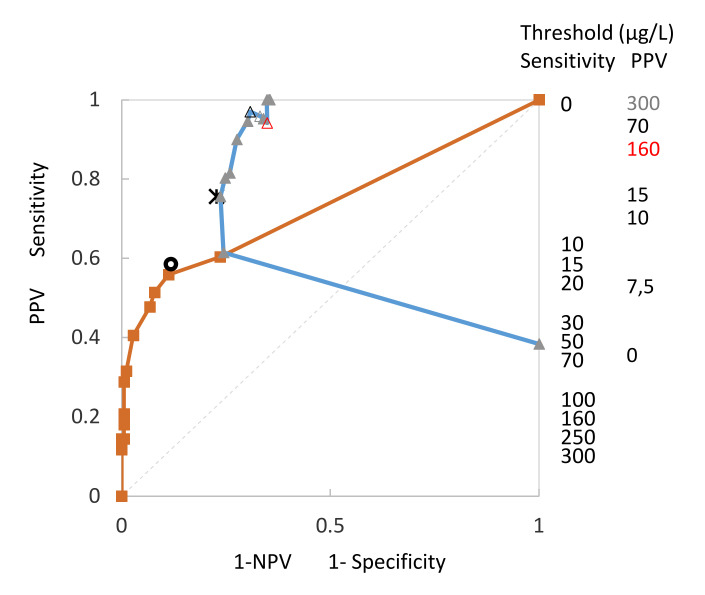
SS-ROC and PV-ROC curves, which were included in a single SS/PV-ROC plot. The curves are calibrated at intervals of the risk score threshold and reveal TPP-asymmetric improper curves, which cross the diagonal from above. The SS-ROC curve is shown as a brown line with squares, the PV-ROC curve as a blue line with triangles. Thresholds on each of the curves can be noted by reading horizontally across from the appropriate column of values on the right-hand side of the plot to the corresponding curve. In addition, the corresponding points calculated from the qualitative UBC^®^ *Rapid* Test predictive value POC test determination for sensitivity/specificity (black circle) and predictive values (black cross) are included in the graph.

Figure 3 shows that all results for sensitivity, specificity and positive and negative predictive results of the risk thresholds can be included in a single SS/PV-ROC plot. The curves are calibrated at intervals of the risk score threshold and reveal TPP-asymmetric improper curves, which cross the diagonal from above. The SS-ROC curve (brown line, squares) is related to all created pairs of true positive rates (TPR) against the false positive rates (FPR) at various threshold value settings for specificity and sensitivity, and the PV-ROC curve (blue line, triangles) consists of all created pairs of PPV and NPV as the threshold for test positivity varied. The threshold numbers on the right side of the graph for sensitivity and PPV are not on a calibrated scale. In addition, the corresponding points calculated from the qualitative UBC^®^ *Rapid* Test predictive value POC Test determination for sensitivity/specificity (black circle) and predictive values (black cross) are included in the graph.

Figure 3 demonstrates that, in contrast to the SS-ROC curve, which (generally) contains an area under the curve (AUC) and a full range from 0% to 100%, the PV-ROC curve has neither an AUC nor a range from 0% to 100%.

**Figure 4:**
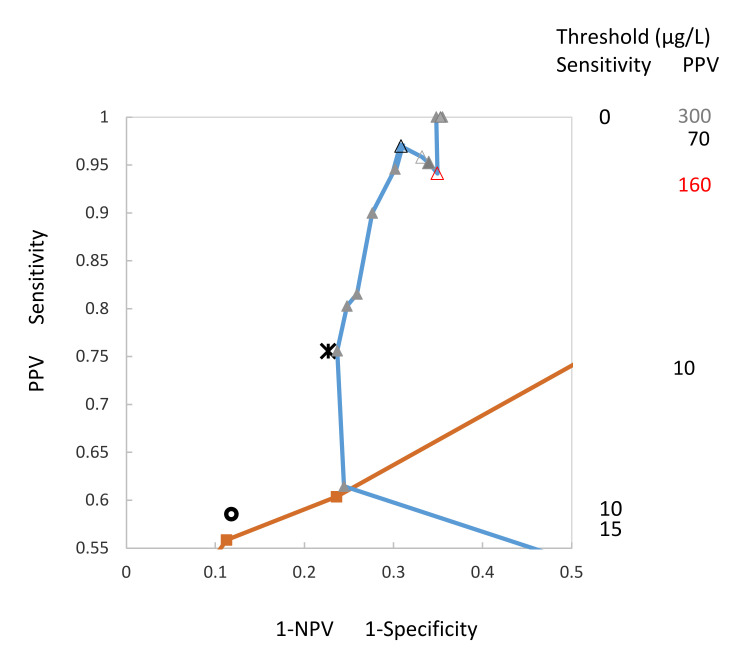
This figure is a section of Figure 3, which can be used to estimate the threshold value concentration for the qualitative test, derived from data for sensitivity (black circle) and PPV (black star), found to be approximately 12.5 µg/L. The graph illustrates decreasing PPV-values starting at a threshold value of 70 (empty black triangle) and ending at 160 (empty red triangle), including the threshold values 100 (empty triangle) and 130 (full triangle). The curves are calibrated at intervals of the risk score threshold. Thresholds on each of the curves can be noted by reading horizontally across from the appropriate column of values on the right-hand side of the plot to the corresponding curve.

In addition to Figure 3, a section of this figure was drawn (Figure 4) to provide a detailed description of the course of the PPV curve, as well as to estimate the threshold concentrations of the visually judged quantitative test. This was done by correlating the threshold values of the quantitative assay to points for the sensitivity/specificity, as well as to those of the predictive values visually in this figure, which led to an approximate threshold value of 12.5 µg/L for both sensitivity and PPV. The graph shows a decrease of PPV starting at a threshold value of 70 (empty black triangle) and ending at 160 (empty red triangle), including the threshold values 100 (empty triangle) and 130 (full triangle).

Regarding the course of the predictive values curve, it is evident that two or more distinct values of PPV can correspond to the same value of NPV and vice versa (Figure 4), indicating a complexity in PV-ROC curves which does not exist in SS-ROC curves. Evaluation of the PV-ROC curve showed an (unexpected) decrease of PPV values at threshold values of 70, 100, 130 and 160.

The sensitivity of the qualitative assay at the threshold of 12.5 µg/L was 58.5%, the specificity was 88.2%, the PPV was 75.6% and the NPV was 77.3%. Figure 3 and Figure 4 show that the values were located close to the respective curves, which confirms a good agreement of results. Visual estimation of the threshold concentration for the quantitative assay seemed to be equivalent to the threshold value range from 10.0 to 12.5 µg/L. At a threshold value of 10 µg/L, the values for the quantitative assay were 55.8, 88.8, 75.6 and 76.3 µg/L. The highest 1-NPV was 0.234 µg/L, close to the NPV threshold of the quantitative assay at a value of 0.227 µg/L.

## 4. Discussion

The SS/PV-ROC plot may become a good tool to judge patient values received from a biomarker determination from a more clinical perspective, within the context of the variable parameters seen in the SS-ROC and PV-ROC curves when both are considered together. Furthermore, it is a valuable approach to characterize quantitative biomarker assays and to compare them with others, including qualitative assays. However, evaluation of such diagnostics seems to be a complex procedure and clinicians will need assistance in learning how to deal with this approach with respect to the complexity of PV-ROC curves. Recently, articles published by Hughes [8] and Benish [9] provided valuable information and aid in dealing with this task (Benish does not discuss PV-ROC). A detailed evaluation of the findings in the presented article would include a discussion via information theory. In this article, which is primarily focused on establishment of the empirical SS/PV-ROC plot, a detailed analysis via information theory is not included. Instead, the reader is referred to the articles of Hughes [8] and Benish [9] who reported on mutual information as a metric for predictive performance for PV-ROC and SS-ROC, respectively.

The investigation presented in this article includes both a study of the potential non-invasive diagnostic approaches for the qualitative and quantitative cancer biomarker UBC^®^ *Rapid* and a study including empirical SS-ROC and PV-ROC curves, using bladder cancer as an example. The results are presented in a single SS/PV-ROC plot for direct characterization, comparison and evaluation of two clinically applied bioassays.

One purpose of this study was to evaluate the clinical usefulness of the UBC^®^ *Rapid* Test for diagnosis of bladder cancer with a focus on patients with non-muscle-invasive high-grade tumors (NMI-HG) of the urinary bladder compared with healthy individuals. The results of the present study show that cytokeratin concentrations determined by the UBC^®^ *Rapid* Test measured by POC reader are statistically significant for patients with bladder cancer compared with healthy controls. Similar results were shown by Pichler et al. [10] and Styrke et al. [11].

The other purpose of this study was to use the results of a quantitative biomarker assay for bladder cancer in order to establish empirical PV-ROC curves and combine them with SS-ROC curves for sensitivity and specificity. Thus, conventional characterization, evaluation and comparability of bioassays could be applied at a broader scale to use this new tool to improve clinical diagnostics.

Concerning sensitivity and specificity for biomarkers, the first ROC curves were published in 1981 by Oehr et al. [12] for different patient groups affected with cancer of the breast, lung, urinary bladder and testis, in comparison to groups of healthy persons or patient groups with benign diseases. Within this first approach, ROC curves were established in order to directly compare different markers or different test systems independent of the correspondent marker concentrations.

Theoretical predictive value ROC curves including a study of the effect of the positivity threshold on the pair of PPV and NPV of tests were first published by Shiu and Gatsonis in 2008 [13], defining the curves mathematically, discussing the geometric patterns of these curves and describing methods for evaluating a test’s predictive performance. According to the authors, it is essential “to study and attempt to characterize the geometric properties of PROC curves before undertaking an investigation of how the curves can be used to evaluate the performance of a diagnostic test” (PROC is a synonym of PV-ROC). To the best of our knowledge, empirical predictive ROC curves have not yet been published for bioassays by other authors, and accordingly this would also be valid for the SS/PV-ROC plot presented in this study.

The underlying investigation of bioassays included the qualitative UBC^®^ *Rapid* urine-based point-of-care (POC) test, which can also be evaluated quantitatively by combining it with a reader system. The first evaluation of both the qualitative and the quantitative UBC^®^ *Rapid* Tests was published by Ritter et al. [2]. In this study, the quantitative UBC^®^ *Rapid* Test showed similar results when compared to the quantitative determination. The sensitivity of the quantitative assay was 55.7%, the specificity was 81.0%, the PPV was 56.7% and the NPV was 80.4%. The results for the quantitative assay were 60.7%, 70.1%, 46.8% and 79.3%, respectively. According to the quantitative assay, the optimal threshold value was calculated to be 12.3 µg/L, using the optimal threshold value obtained by receiver operating characteristic analysis for the quantitative assay according to the highest Youden index. However, a threshold for the qualitative assay was not known and could not be included for comparison. For comparing two assays, technically using the same POC test as a base for antigen detection, it would be optimal to use the same threshold value because at different thresholds the deviation of the results will normally increase, as can be seen according to threshold changes in the SS/PV-ROC plot in Figure 3. Without knowledge of both threshold values, however, the resulting diagnostic values can only reflect the degree of deviation, but such data cannot be interpreted in the same way as results from a direct comparison under (broadly similar) defined conditions.

With respect to the study presented in this article, the resulting diagnostic values found by comparison of the qualitative and quantitative assays are much closer to each other. Sensitivity of the qualitative assay was 58.5%, specificity 88.2%, PPV 75.6% and NPV 77.3%. In comparison, at the threshold concentration value of 10 µg/L, sensitivity of the quantitative assay was 55.8%, the specificity was 88.8%, the PPV was 75.6%, and the NPV was 77.3%. These results give evidence that both assays show a high agreement. The comparison could be made by prior estimation of the threshold by use of the SS/PV-ROC plot, and the outcome of this approach might be taken as an example for its utility.

The reason for including the thresholds for the resulting values of sensitivity and PPV in Figure 3 and Figure 4 is that developing ROC curves from cut-off distribution curves for sensitivity/specificity or predictive values involves loss of information about the threshold concentrations. Adding the threshold values into the SS/PV-ROC plot again is regarded as important supplementary information which supports the reader in understanding and interpretation of SS/PV-ROC plots. It is important to know how the thresholds for the curves change and that the changes are not gradual but dynamic. As for publications using the SS/PV-ROC plot, it is recommended to use this approach.

The optimal threshold is presently calculated by most authors of tumor marker studies according to the highest Youden index. Ritter et al. [2] published an optimal cutoff at ≥12.3 µg/L. Styrke et al. [11] calculated an optimal threshold value at ≥8.1 μg/L, resulting in a sensitivity of 70.8%, specificity of 61.4%, PPV of 71.3% and NPV of 60.8%. Pichler et al. [10] reported the best cutoff at a threshold value of 6.7 ng/mL. The sensitivity, specificity, PPV and NPV of the visually evaluated qualitative UBC^®^ *Rapid* Test were 61.3%, 77.3%, 65.5% and 73.9%, respectively. For the quantitative UBC^®^ *Rapid* Test, sensitivity, specificity, PPV and NPV were 64.5%, 81.8%, 71.4% and 76.6%, respectively. This accumulation of different optimal threshold values in diagnostics and follow-up of bladder cancer patients seems to be confusing. The reason for differences in optimal threshold values may be related to different clinical states of the included patients and/or controls.

To find the appropriate threshold, regarding the clinical situation for requesting an examination of a patient, deriving threshold decisions from an SS/PV-ROC plot might be an alternative solution. At present, the calculation of the highest Youden index disregards the predictive values, and the low specificity of the published optimal values might lead to an increased number of false positive values, which could involve unnecessary invasive diagnostics in clinical follow-up of patients with suspected cancer.

Concerning the establishment of the SS/PV-ROC plot for the present study, an artifact appears to have evolved when plotting the empirical PPV-ROC at high values in a study with low case numbers. As illustrated in Figure 4, there was an (unexpected) decrease of PPV values at threshold values of 70, 100, 130 and 160. This was due to the fact that in case of all calculations for this threshold range there was always only a single case result for false positive values (FPR) in the calculations for PPV. There was no effect on NPV, since FPR values are not included in the calculations. In case a study includes a higher number of cases, this artifact effect might decrease or disappear. Specificity results regarding the mentioned FPR values were not markedly affected because the results for specificity were 99.4% in all cases.

The present study of the SS/PV-ROC plot is regarded as a first step, and application of this approach in daily clinical work is still regarded a goal. As we understand more about the characteristics of SS/PV-ROC plots, there will be opportunities for the re-analysis of existing datasets in order to gain a more detailed understanding of the operation of risk thresholds. The study by Styrke et al. [11] is one such example.

At present, evaluation of empirical PV-ROC curves remains a difficult task. Unlike the SS-ROC, the form of PV-ROC is dependent on prevalence of cases in the dataset, and this has some impact on the extent to which any particular SS/PV-ROC can be generalized. PPV and 1−NPV increase as prevalence increases [8]. With respect to urinary bladder cancer, prevalence is known to differ in men and woman. Here, for the purpose of illustration, we treated the data as homogeneous. However, we note that, where sources of heterogeneity can be identified statistically within a dataset, this might call for separate PV-ROC curves for the different sub-sets. This is beyond the scope of the present article but worth noting as a topic for further research.

Support is necessary to understand the information theoretic perspective on evaluation, as well as to provide recommendations with a view to aiding understanding and interpretation of the sometimes-complex patterns generated by PV-ROC curves, their correlations with SS-ROC and their correlations to other related statistical methods, including estimation of prevalence and the leaf plot. Furthermore, to obtain agreement on a standardized PV-ROC curve evaluation, it is important to make future empirical studies by different authors or institutions comparable. Recently published articles [8,9] will help to develop this new path relating to the diagnostics of bioassays for cancer and provide a base in other fields of science for general application of the SS/PV-ROC plot.

## Figures and Tables

**Table 1:** Characteristics of bladder cancer patients.

	Status	*n* (%)
Tumor stage	pTa	61 (55%)
	pT1	14 (12.6%)
	pT2	23 (20.7%)
	pT3	9 (8.1%)
	only CIS	3 (2.7%)
	n.a.	1 (1%)
Grading	G1	26 (23.4%)
	G2	58 (52.3%)
	G3	24 (21.6%)
	G4	1 (0.9%)
	n.a.	2 (1.8%)
EORTC—Risk	Low risk	9
	Intermediate risk	43
	High risk	27
	n.a.	32
Number of tumors in bladder	1	50
	2–7	39
	≥8	8
	Not specified	14
Diameter of tumors in the bladder	Ø < 3 cm	56
	Ø > 3 cm	43
	n.a.	12
Primary vs. Recurrent tumors	Primary	58
	Recurrent	52
	n.a.	1
Number of recurrence	1	25
	2	6
	3	6
	≥4	14
	n.a.	1
Gross hematuria	yes	66
	no	45
Alguria	yes	33
	no	78

Explanation of abbreviated medical terminology. Tumor stage: The extent of a cancer in the body. Staging is usually based on the size of the tumor, whether lymph nodes contain cancer and whether the cancer has spread from the original site to other parts of the body; pTa tumors are those neoplasms that are confined to the epithelial layer of the bladder; pT1 tumors are those that invade into the subepithelial connective tissue; CIS (Carcinoma in situ) is a “flat tumor” of the epithelial layer; T2–T4 are muscle-invasive tumors. Tumor grade (G): A description of a tumor based on how abnormal the cancer cells and tissue look under a microscope and how quickly the cancer cells are likely to grow and spread; GI are cancer cells that resemble normal cells and are not growing rapidly; GII are cancer cells that do not look like normal cells and are growing faster than normal cells; GIII are cancer cells that look abnormal and may grow or spread more aggressively. Gross hematuria: Blood in the urine that can be seen with the naked eye. Alguria: Burning sensation when voiding.

**Table 2:** Description of study data.

		Cancer	NED	NMI-LG	NMI-HG	MI-HG	Control	Total
		(*n* = 111)	(*n* = 32)	(*n* = 56)	(*n* = 22)	(*n* = 33)	(*n* = 146)	(*n* = 289)
Age (years)								
	Mean (SD)	71.19 (11.46)	68.78 (13.14)	70.80 (12.35)	72.45 (9.41)	73.23 (9.31)	69.61 (11.94)	70.39 (11.71)
	Median	74	70.5	72	75	74	71.5	73
	Range	26 to 92	46 to 88	26 to 92	51 to 92	53 to 88	33 to 93	26 to 93
	*n*	111	32	56	22	33	146	289
Gender (M, F)	*n* (%)							
F		28 (25.23)	7 (21.88)	14 (25.00)	2 (9.09)	12 (36.36)	46 (31.51)	81 (28.03)
M		83 (74.77)	25 (78.12)	42 (75.00)	20 (90.91)	21 (62.64)	100 (68.49)	208 (71.93)
UBC (µg/L)								
	Mean (SD)	53.64 (87.93)	12.37 (11.40)	44.61 (81.98)	109.26 (115.76)	70.85 (97.12)	7.58 (14.00)	30.37 (66.66)
	Median	10.5	6.45	6.15	59.4	20.7	5	5
	Range	5 to 300	5 to 56.5	5 to 300	5 to 300	5 to 300	5 to 166	5 to 300
	*n*	111	32	56	22	33	146	289

Explanation of abbreviated medical terminology: NED, No evidence of disease according to the Guidelines on Non-Muscle-Invasive Urothelial Carcinoma [5,6]; NMIBC, Non-Muscle-Invasive Bladder Cancer; (TaT1 or carcinoma in situ (CIS); NMI-LG, Non-Muscle-Invasive Low-Grade (Bladder Cancer); NMI-HG, Non-Muscle-Invasive High-Grade (Bladder Cancer); MI-HG, Muscle-Invasive High-Grad (Bladder Cancer).
